# New discovery on the nematode activity of aureothin and alloaureothin isolated from endophytic bacteria *Streptomyces* sp. AE170020

**DOI:** 10.1038/s41598-022-07879-w

**Published:** 2022-03-10

**Authors:** Min-Kyoung Kang, Jong-Hoon Kim, Min-Jiao Liu, Chun-Zhi Jin, Dong-Jin Park, Junheon Kim, Bong-Hyun Sung, Chang-Jin Kim, Kwang-Hee Son

**Affiliations:** 1 Industrial Biomaterial Research Center, Korea Research Institute of Bioscience and Biotechnology (KRIBB), Daejeon, 34141 South Korea; 2 Synthetic Biology and Bioengineering Research Center, Korea Research Institute of Bioscience and Biotechnology (KRIBB), Daejeon, 34141 South Korea; 3 Department of Bio-Molecular Science, KRIBB School of Bioscience, Korea University of Science and Technology (UST), 217 Gajeong-ro, Yuseong-gu, Daejeon, South Korea; 4 Forest Insect Pests and Diseases Division, National Institute of Forest Science, Hoegi 57, Dongdaemun, Seoul, 02455 South Korea

**Keywords:** Biophysics, Microbiology, Plant sciences

## Abstract

Endophytic bacteria, a rich source of bioactive secondary metabolites, are ideal candidates for environmentally benign agents. In this study, an endophytic strain, *Streptomyces* sp. AE170020, was isolated and selected for the purification of nematicidal substances based on its high nematicidal activity. Two highly active components, aureothin and alloaureothin, were identified, and their chemical structures were determined using spectroscopic analysis. Both compounds suppressed the growth, reproduction, and behavior of *Bursaphelenchus xylophilus*. In in vivo experiments, the extracts of strain *Streptomyces* sp. AE170020 effectively suppressed the development of pine wilt disease in 4-year-old plants of *Pinus densiflora*. The potency of secondary metabolites isolated from endophytic strains suggests applications in controlling *Bursaphelenchus xylophilus* and opens an avenue for further research on exploring bioactive substances against the pine wood nematode*.*

## Introduction

Pine trees are one of the most important tree species in global ecosystems. They are not only a predominant component of natural reserves, parks, and urban ornamental landscapes but are also a valuable source of high-value timber carpentry items. The most serious threat to pine forests worldwide is pine wilt disease (PWD) caused by the pine wood nematode (PWN), *Bursaphelenchus xylophilus*. *B. xylophilus* is a quarantined plant parasitic nematode of the Aphelenchoidoidea superfamily and belongs to clade 10 tylenchida^1^. Native to North America, it was first introduced to Japan at the beginning of the twentieth century^2^. Since then, PWN has spread further to China, South Korea, Portugal, and thereafter to Europe^3,4^. When pine trees are infected with pathogenic PWN, some dynamic host responses are induced by the nematode infection, such as the generation of superoxide anions, the development of vacuoles in ray parenchyma cells to capacity, and a dramatic increase in lipid peroxide levels, resulting in host mortality^5^. PWD, a disease that has reportedly caused significant damage to forestry, ecologies of the affected countries, and local economies, is becoming a matter of concern in several continents.

The current means of controlling PWNs rely on chemical nematicides, which are expensive, inefficient and have adverse impacts on the environment and human health^6^. Indeed, the use of beneficial bacteria and bacterial nematicides has spawned intense interest in the development of safer alternatives for PWN management^7,8^. However, many problems remain regarding the bacterial control of nematodes, including the relative scarcity of nematicidal bacteria. Only a few bacteria have been reported to possess activity against nematodes and to show potential for the biological control of nematodes^9–11^. Identifying novel nematicidal bacteria has become urgent for the bacterial control of nematodes and is of vital practical and economic significance.

Endophytic bacteria that reside in the tissues of living plants and establish a symbiotic relationship with the host are relatively unstudied and potent sources of novel natural products for application in medicine, industry, and agriculture^12–14^. Several reports have investigated bacterial endophytes as possible agents against PWNs^7,15^. These bacteria kill nematodes by different mechanisms, including by mobilizing plant defenses and producing an array of secondary metabolites and enzymes.

The community of endophytic bacteria in *Pinus* spp. has been investigated using several methods, such as cultivation-based methodologies, biochemical characterization of the isolates by BIOLOG phenotypic assay^16^, and molecular approaches^17–19^. Strains belonging to the genera *Bacillus*, *Paenibacillus*, and *Pseudomonas* were isolated from several pine tree species, and some of these bacteria were found to produce siderophores^17^ or have the ability to fix nitrogen^18,19^. However, bacteria associated with *Pinus* spp. have rarely been explored with regard to their nematicidal activity against *B. xylophilus*^20^. Therefore, the objectives of this study are (i) to isolate the nematicidal substances from *Streptomyces* sp. AE170020, an endophytic bacteria isolated from root tissues of pine tree; (ii) to investigate the effects of active substances on *B. xylophilus*; and (iii) to evaluate the disease control efficacy of the strain as a biocontrol agent in vivo.

## Materials and methods

### General instruments

The ^1^H nuclear magnetic resonance (NMR) and ^13^C NMR spectra were obtained on a Bruker Biospin Avance 400 spectrometer with tetramethylsilane (TMS) as the internal standard. Electrospray ionization–mass spectrometry (ESI–MS) was performed on an Agilent 6430 LC/MS/MS and a 1100 LC/MS spectrometer. The high-performance liquid chromatography (HPLC) system comprised a Hitachi Model L-2130 pump, L-2400 UV detector, and YMC J’sphere ODS H-80 column (4 mm, 20 × 150 mm, YMC Co., Ltd).

### Chemicals

Highly pure, chemical-grade n-Hexane, chloroform (CHCl_3_), ethyl acetate (EtOAc), *n*-buthanol (*n*-BuOH), and acetone were purchased from Samchun Pure Chemical Co., Ltd., Korea. Dimethyl sulfoxide (DMSO, 99.9%, spectrophotometric grade), trifluoroacetic acid (TFA, for HPLC, ≥ 99.0%), proteinase K, abamectin, propylene glycol monomethyl ether (PGME), propylene glycol (PG), thiobarbituric acid, trichloroacetic acid, and Na_2_CO_3_ were obtained from Sigma–Aldrich Chemie GmbH, Steinheim, Germany. MeOH and water for HPLC analysis were purchased from Duksan Pure Chemicals Co., Ltd., Korea. Folin-Ciocalteu’s phenol reagent was obtained from Merck KGaA, Darmstadt, Germany. Tris buffer and gallic acid were purchased from Bioneer Corp and MP Biomedicals, Korea, respectively. Alloaureothin and aureothin used in the experiment were separated by prep TLC, and then used as a compound with a purity of 90% or more by HPLC.

### Isolation and molecular identification of Strain AE170020

Strain AE170020 was isolated from root tissues of pine tree samples collected from Jinju (J, 35º12.319′ N, 128º10.49ʹ E, altitude, 91 m), Korea. Briefly, plant samples were washed with an ultrasonic step (160 W, 30 min) to remove the surface soils and epiphytes. The washed plants were put into sterilized Petri dishes with aseptic filter paper to remove the surface water. After drying, plants were subjected to a seven-step surface sterilization procedure: immersion in 0.1% Tween 20 for 1 min, followed by a 6-min wash in 5% sodium hypochlorite, a 10-min wash in 2.5% sterilized sodium thiosulfate, three washes in sterile water, a 4-min wash in 70% ethanol, five washes in sterile distilled water, and a final rinse in 5% sodium bicarbonate for 10 min. Samples were then put into 2 ml sterile tubes and grinded using liquid nitrogen and then put onto TWYE media (0.25 g Yeast extract, 0.5 g K_2_HPO_4_, agar, 1 L DW). After 6 weeks of inoculation, the isolate was selected and purified. Genomic DNA was extracted, and the 16S rRNA gene was amplified using polymerase reaction. The resulting 16S rRNA gene sequence was compared with the curated sequences from EzBioCloud (http://www.ezbiocloud.net/) and a phylogenetic tree was built with the neighbor-joining method.

### Acquisition of pine wood nematode (PWD)

*Bursaphelenchus xylophilus* were reared on a lawn of *B. cinerea* cultured on PDA medium in the dark at 25 °C for 7 days; nematodes were then extracted using the Baermann funnel method for bioassay^21^.

To obtain bacteria-free PWNs, nematodes were extracted and transferred to Petri dishes containing sterile distilled water to allow egg laying for 3 h. Most eggs adhered to the bottom surface of the Petri dish and were gently washed with distilled water many times to remove the hatched nematodes. Then, 15% H_2_O_2_ was added into the Petri dish, which was rinsed for 60 min at 25 °C; next, the Petri dish was washed with sterile water three times. Eggs were then resuspended in sterile distilled water and allowed to hatch. The PWN J2_S_ that were almost synchronized after 24 h at 25 °C were obtained and used for mortality assays. The J2_S_ were transferred onto a culture of *B. cinerea* on a PDA plate*,* and J3_S_ and J4_S_/adults were collected at 30 h and 78 h after feeding re-initiation, respectively.

### Detecting nematicidal activity of strain *Streptomyces* sp. AE170020

The strain was inoculated into the GSS broth (20 g glucose; 10 g soluble starch; 25 g soybean meal; 1 g beef extract; 4 g yeast extract; 2 g NaCl; 0.25 g K_2_HPO_4_; 2 g CaCO_3_, and 1 L DW) for 36 h. Two percent of the seed was transferred to a 500 mL flask containing 50 mL of GSS broth and incubated on a rotary shaker at 150 rpm at 28 °C for 1–10 days. The culture of strain AE170020 was then centrifuged at 13,000 rpm for 10 min to separate the broth and cells. The resultant supernatant was aseptically transferred to sterile tubes and used for a nematicidal bioassay, as described by Gao et al., with some modification^22^. Briefly, 50 μL of the supernatant and bacteria-free J2s of *B. xylophilus* (n = 30–40) specimens were added to each well of a 96-well tissue culture plate. Later, 100 μg mL^−1^ streptomycin was also added to each well to inhibit bacterial contamination, and GSS broth was used as a control.

### Detecting the stability of nematicidal activity of *Streptomyces* sp. AE170020 culture

The stability of strain AE170020 was detected following the method of Gao et al.^22^. The supernatant of strain *Streptomyces* sp. AE170020 was treated with 20 mg mL^−1^ of proteinase K at 37 °C for 30 min or boiled at 100 °C for 5 min. Thereafter, the nematicidal activity of the supernatant was detected. This supernatant was also adjusted to pH values from 1.0 to 11.0 by HCl or NaOH solution and incubated for 2 h; the nematicidal activity of the supernatant was then detected after the pH value was re-adjusted to 7.0.

### Morphological characteristics of selected strain

To investigate morphological characteristics, strain AE170020 strain was streaked on a Bennett's agar medium. The strain was cultured at 28 °C for 14 days color of the mycelium of the metal-bearing strain were observed using a scanning electron microscope (FEI Quanta 250 FEG; FEI, US). The spore morphology was photographed as follows. Spore and spore form, size of strain AE170020 were determined on M3 agar media (10 g glucose, 10 g soytone, 20 g soluble starch, 3 g CaCO_3_, 0.2 g FeSO_4_⋅7H_2_O, 20 g agar and 1 L DW). After incubation at 28 °C for 10 days, the morphological characteristics of the strain AE170020 strain were observed under an optical microscope (Nikon Labophot-2).

### Fermentation, extraction, and purification of nematicidal substances from *Streptomyces* sp. AE170020 culture

Strain cultures were prepared as described above and centrifuged at 6000 rpm for 30 min. The collected supernatant was then sequentially extracted with an equivalent volume of *n*-hexane, CHCl_3_, EtOAc, and *n*-BuOH. The mycelium of strain AE170020 was extracted with acetone, sonicated for 30 min, and kept overnight. All extractions were performed thrice. Different solvent layers were concentrated in vacuo to obtain the dry extracts. The nematicidal activity of the extracts was determined: 2 μL of the solvent extract of *Streptomyces* sp. AE170020 was dissolved in DMSO and added to the 96-well plate, containing approximately 40–50 J2s nematodes in 98 μL of sterile water. The final concentrations of solvent extracts were 0.0625, 0.1250, 0.2500, 0.500, and 1.000 mg mL^−1^, and 2 μL of DMSO was used as a control. Acetone extracts, which showed the highest activity against PWN, were used for further purification.

The fractions extracted by acetone were applied on a silica gel column and eluted with a stepwise *n*-hexane/acetone gradient of increasing polarity. Fractions were checked on a TLC plate, and those with similar TLC patterns were pooled for nematicidal activity testing. One of the fractions that showed high nematicidal activity was applied on a Sephadex LH-20 column and eluted with MeOH. Nematicidal activity of subfractions was tested and combined as above. Further, active subfractions were purified with preparative TLC plates, and two active compounds were obtained. HPLC (TKS gel ODS, 250 × 4.6 mm, 5.0 µm, 25 cm) with a gradient solvent system of aqueous 85% MeOH, containing 0.04% TFA, as a mobile phase was used to check the purity of the compounds. The peaks of the two active compounds were confirmed at 22 min. They were named **1** and **2**, respectively (Fig. 4A,B).

### Different developmental stages of *B. xylophilus* mortality assay

Two of the most potent purified compounds, alloaueohtin and aureothin, were further explored for their effects on the different stages, reproduction, growth, and behavior of *B. xylophilus*. Different developmental stages of nematode were prepared as previously described, and J2s were exposed to the two compounds at final concentrations of 0.05, 0.10, 0.20, 0.50, 1.00, 2.00, and 4.00 μg mL^−1^; J3s and J4s/adults were exposed to concentrations of 0.25, 0.50, 1.00, 2.50, 5.00, and 10.00 μg mL^−1^. The commercial nematicide, abamectin, at the same concentrations and DMSO were used as positive and solvent controls, respectively.

The treated plates were stored in the dark at 25 °C, and the mortality of PWNs was recorded after 24-h treatment by visualization under a light microscope (Nikon, SMZ-U). The LC_50_ values of active compounds at different life stages were estimated. Mortality was defined based on the observation of motility, a visibly moving nematode was marked as alive, and nematodes that failed to respond after several touches were marked as dead. The bioassays were performed three times in triplicate. Mortality was calculated according to the following formula: mortality (%) = dead juveniles/total juveniles × 100.

### Effects of compounds on the locomotion behavior of *B. xylophilus*

In liquids, a single movement of the stylet knob forwards and then backwards to its original position was called “thrashing”^23^. To assess the impact of alloaureothin and aureothin on *B. xylophilus* mortality, thrashing of J3s nematode was assayed. Thrashing rate was scored for 1 min at the following time points: 2, 6, and 24 h in the presence of two compounds at the indicated concentrations (1.0, 2.5 and 5.0 μg mL^−1^). Control assays were conducted in the presence of DMSO or abamectin, and all assays were conducted at 22 °C. Two trials were performed for this experiment, and six numbers of surviving nematode in each treatment were used to test the thrashing behavior.

### Nematode population inhibition assay

A population inhibition assay was conducted according to the method described by Cheng et al.^23^ with some modification. Briefly, adult nematodes (~ 500) were initially treated with alloaureothin and aureothin at concentrations of 0.5 and 1.0 μg mL^−1^, and incubated for 24 h at 22 °C. After 24 h, 10 female and male nematodes were randomly selected and placed on a PDA plate fully covered with *B. cinerea* and allowed to grow. When the *B. cinerea* has been completely consumed by nematodes in control plates, nematodes were extracted from plates using distilled water. The recovered nematodes were then serially diluted and numbers of nematodes in 100 μL suspensions were counted. The reproduction rates (P_f_/P_i_, P_f_, final nematode population; P_i_, initial nematode population) were calculated. The experiment was conducted twice, with three replicates per treatment.

### Nematode hatch inhibition assay

Nematode eggs were obtained as above and used for the embryonic lethality tests. Experiments were conducted using established procedures^23^. Egg suspensions were exposed to alloaureothin and aureothin at final concentrations of 20, 15, 10, 5, 2, 1, 0.5, and 0.1 μg mL^−1^. Two microliters of DMSO and abamectin at the same concentration were served as negative and positive, respectively. Plates were incubated at 22 °C and nematodes of the J2s stage were scored after 48 h. The hatching rate was calculated according to the following formula: Hatching rate (%, HR) = [juveniles/(eggs + juveniles) × 100]. Each treatment was repeated in three wells, and the experiment was repeated three times.

### Egg deposition assay

One-day-old female nematodes in the presence of alloaureothin, aureothin or abamectin (5 µg mL^−1^) were used in the egg deposition assay according to an established protocol^23^_._ *B. xylophilus* (about 100 numbers) were treated with compound for 24 h in the 96-well plates. Approximately 10 male and 10 female nematodes were then selected randomly and transferred to Petri dishes and allowed to lay eggs for 24 h. The eggs laid by a single female were then recorded and the nematodes inside the female body were also imaged. The experiments were performed two times with six repetitions.

### Suppression of pine wilt disease under pot condition

Injection of active substances into pine trees was performed to evaluate the control efficacy in vivo, as previously described^24^. The pot experiments were conducted in a greenhouse at Korea Research Institute of Bioscience and Biotechnology (Daejeon, Korea) and *Pinus densiflora* were transferred to a greenhouse for at least 1 month prior to the pot test and watered every other day and fertilized if necessary. The average temperature of the greenhouse was 25 ± 5 °C. Acetone extracts of strain AE170020 were prepared as described above and dissolved using DPPT solvent (20% DMSO, 20% propylene glycol monomethyl ether, 50% propylene glycol, and 10% Tween-20). The solution of abamectin in DPPT solvent served as a positive control. Holes were made in the trunk of pine trees (average height, 124 cm; average basal diameter, 1.4 cm) by using an electric drill about 5 cm above the ground level. Then, 200 μL of strain extract was injected into holes at different concentrations, and holes were immediately covered with parafilm. The final concentration of the acetone extract was 7.2 mg, 3.6 mg, and 1.8 mg per tree and abamectin was 3.6 mg per tree. Control plants were injected with the DPPT solution. Inoculations of *B. xylophilus* were performed 1 week after the injection of chemicals in the trunk. *B. xylophilus* were reared on *B. cinerea* and extracted; they were then washed with distilled water at least five times to remove any fungal hyphae present. Final nematode density in the suspension was adjusted to about 20,000 nematodes per mL. A hole in the trunk of each pine tree was made approximately 20 cm above the soil. A 0.1 mL aliquot of the nematode suspension (containing about 2000 nematodes: a mixture of adults and juveniles) was pipetted into the holes and the holes were covered with parafilm. Control plants were also wounded but distilled water was used instead of the PWN solution. Pine trees were watered three times a week. Three runs with five replicates for each treatment were conducted. External symptoms of pine trees were visually assessed at 15, 30, 45, and 60 days after the inoculation and results were recorded at 60 days.

### Sample collection and processing

Sixty days after inoculation, three pine trees from each treatment in one trial were randomly selected and collected. Plants were cut immediately above the ground. Needles and the twigs were removed, and the stems were chopped into small pieces. Stems were immediately weighed and used for nematode quantification. The remaining stems and leaves were stored at − 80 °C for chemical analysis.

### Nematode quantification in inoculated trees

The nematode population was measured using established procedures^25^. Five grams of the stem pieces were weighed and transferred into gauze equipped in the upper part of a 50 mL falcon tube. The plant tissues were then immersed in water for 24 h, and after that the gauze and plants were gently removed from tubes and allowed to precipitate for 12 h. Nematodes were mostly located at the bottom of the falcon tube after the precipitation and the upper water was removed to make a final volume of 10 mL. Then, 100 μL of the extracted nematodes was transferred into 96-well tissue plates, and live nematodes were counted under a light microscope. Nematode density was expressed as the number of nematodes in the stem per gram of wood. The data of each treatment were generated from three trees and obtained in triplicate.

### Chemical analysis

Chemical analyses were performed on leaf tissues (total chlorophyll, total polyphenolics, and lipid peroxidation) and stem tissues (water content). Leaves collected as previously described were used for chlorophyll quantification. The extraction and determination of the chlorophyll content were performed as per the method described by^25^. Briefly, needles were homogenized with liquid nitrogen, and samples (about 0.1 g) were extracted with 10 mL of cold acetone/Tris buffer (80:20 vol:vol, pH = 7.8). The samples were incubated at 4 °C for 72 h, and the tubes were centrifuged at 13,000 rpm for 5 min. The absorbances were then recorded at 537, 647, and 663 nm in a UV/visible spectrophotometer (Pharmacia Biotech, Ultraspec 3000). The amount of chlorophyll was calculated as follows: total chlorophyll concentration in the extract (mmol mL^−1^) = (0.001373*A*_663_ – 0.000897*A*_537_ – 0.003046*A*_647_) + (0.02405*A*_647_ – 0.004305*A*_537_ – 0.005507*A*_663_).

The concentration of total soluble phenolics was determined using the protocol described by Ainsworth et al.^26^. Needles were ground with liquid nitrogen; about 100 mg of leaves was extracted with 95% methanol and sonicated for 15 min. Samples were then gently shook and allowed to react overnight in the dark at room temperature. Later, 200 μL of 10% (vol/vol) Folin–Ciocalteu’s phenol reagent was added to 100 μL of the methanolic extract. The solution was mixed thoroughly and allowed to stand for 5 min, after which 800 μL of 700 mM Na_2_CO_3_ was added, and the tubes were incubated at ambient room temperature for 2 h. Next, 150 μL of each sample solution was loaded on a 96-well microplate and the absorbance was measured at 760 nm with a spectrophotometric microplate reader (VERSAmax™). The total phenolic concentration was calculated from the gallic acid calibration curve. Data were expressed as gallic acid equivalents (GA)/g of extracts averaged from each treatment.

To gain insights about plant oxidative stress and cell damage, lipid peroxidation was measured. The level of lipid peroxidation in tissues was determined in terms of the malondialdehyde (MDA) content by the method of Nunes et al.^25^. After homogenization with liquid nitrogen, about 0.1 g of leaf sample was extracted with 10 mL 0.5% thiobarbituric acid in 20% trichloroacetic acid. The sample was then vortexed thoroughly and incubated at 100 °C for 30 min. Next, reaction solutions were immediately transferred to ice for 5 min and then centrifuged at 4,000 rpm for 15 min. The absorbance was monitored at 450, 532, and 600 nm in a UV/visible spectrophotometer. Calculation of MDA was based on the following formula: MDA concentration (μmol L^−1^) = 6.45(*A*_532_ – *A*_600_) – 0.56*A*_450_.

Specific water content was used as a proxy for water stress, which is usually associated with PWD. For water content determination, each wood sample was oven dried for 72 h, and the relative water content was evaluated through the following formula: relative water content (%) = (fresh weight – dry weight)/fresh weight × 100. All the chemical data were obtained from six trees and three samples per tree.

### Statistical analysis

Data analyses were performed using SPSS 18.0 software (SPSS Inc.), and the LC_50_ values were determined via a probit analysis. Values are expressed as mean ± standard deviation (SD) unless indicated otherwise. The data were compared using analysis of variance (ANOVA) followed by a *post-hoc* test, as appropriate. Significant differences were determined according to thresholds of *p < 0.05; **p < 0.01, and ***p < 0.001. All charts and figures generated in this study were constructed using GraphPad Prism version 8.0.2.

## Results

### Morphological characteristics and phylogenetic analysis

**Figure 1:**
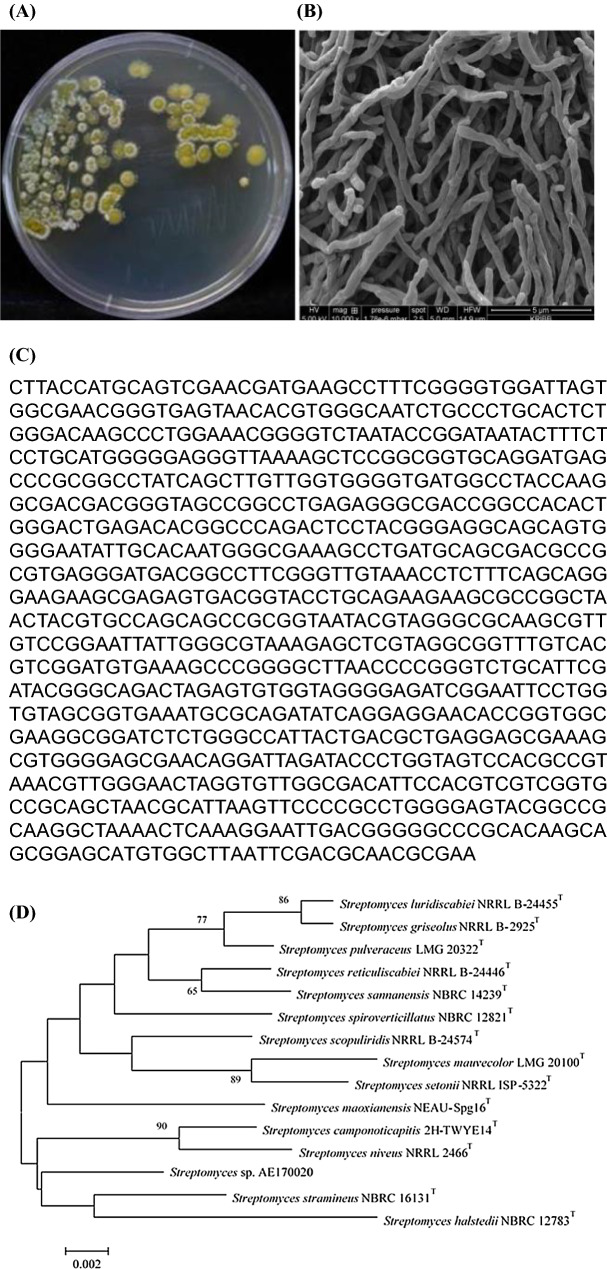
(**A**) Morphological characteristics of active strains on Bennett’s agar media. (**B**) Observation of spore morphology of *Streptomyces* sp. AE170020 under scanning electron microscope. (**C**) Phylogenetic trees based on 16S rRNA gene Sequence of *Streptomyces* sp. AE170020. (**D**) Phylogenetic analysis showing the relationship between strain *Streptomyces* sp. AE170020 and the related species. The tree was generated by neighbor-joining method of 16S rRNA gene sequences. Numbers of nodes are bootstrap values based on 1000 resampling.

The morphological characteristics of the selected active strains AE170020 was analyzed. Morphological characteristics were observed by culturing three strains in M3 agar media, a basic medium for actinomycetes. The AE170020 stain formed circular colonies with white/Gray aerial mycelium and yellow substrate mycelium (Fig. 1A). When strain AE170020 was observed using scanning electron microscopy, retinaculiaperti type spore chains with smooth spore surfaces 0.5–1 µm in size were observed (Fig. 1B).

The identification of 16S rRNA gene sequence using EzTaxon server revealed that strain AE170020 belongs to the genus *Streptomyces* and it shared the highest (98.4%) sequence similarity with *Streptomyces stramineus* (Fig. 1C). The neighbor-joining phylogenetic trees analysis showed that strain AE170020 lies in a subclade in the tree with species of genera *Streptomyces* (Fig. 1D). *Streptomyces* sp. AE170020 was deposited with the Korean Collection for Type Cultures (KCTC) with accession number "KCTC14792BP".

### Characterization of nematicidal activity of *Streptomyces* sp. AE170020

**Figure 2:**
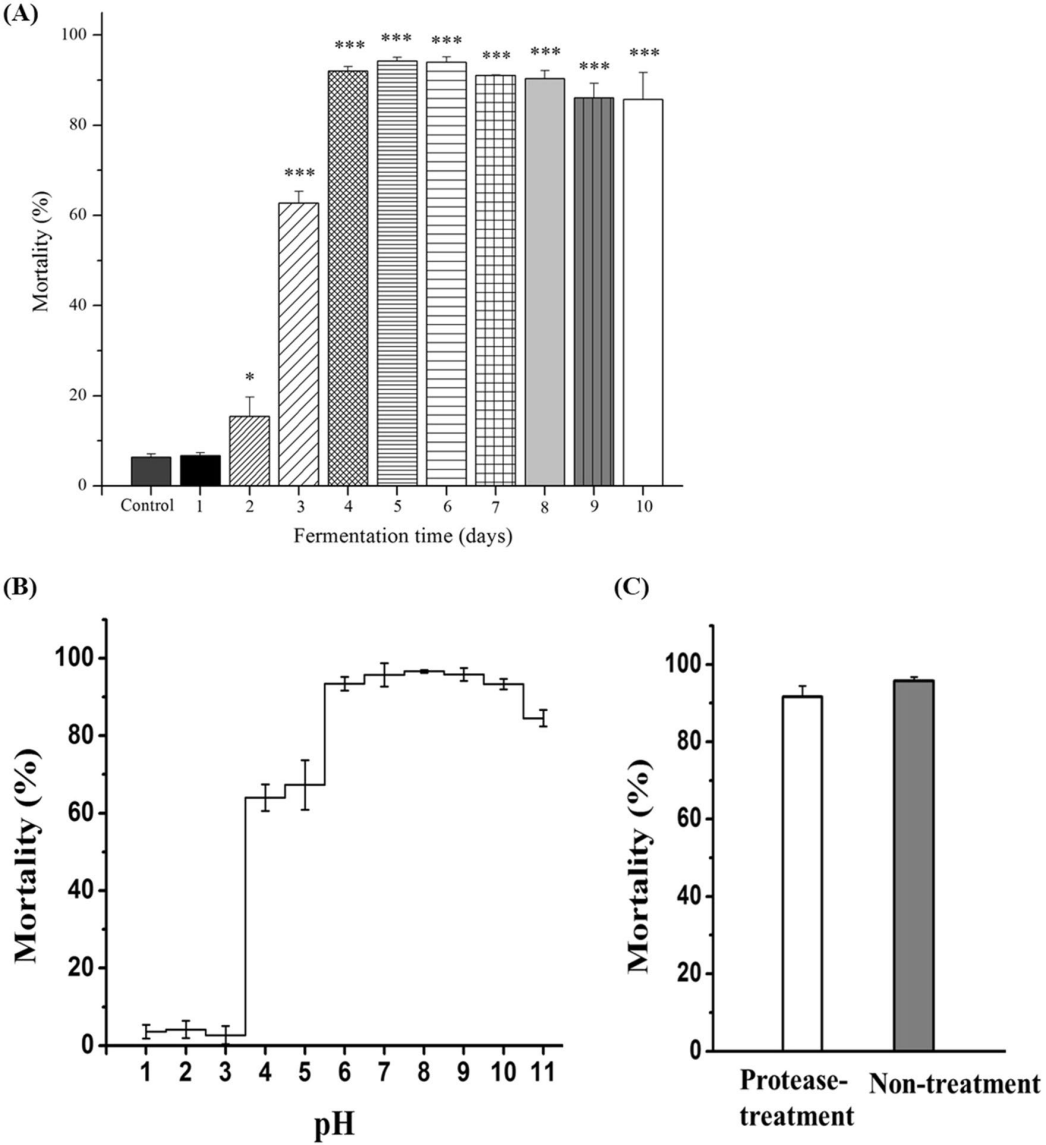
(**A**) Effect of the fermentation time on the nematicidal activity against *Bursaphelenchus xylophilus* of *Streptomyces* sp. AE170020. Nematicidal activity was assessed by calculating the average mortality rate of nematodes treated with *Streptomyces* sp. AE170020 fermentation supernatant. Mortality was calculated 24 h after the treatment. Control, filtrate of GSS medium. Values are expressed as the mean ± SD of two independent experiments with three replicates. Nematicidal activity of *Streptomyces* sp. AE170020 supernatants under different conditions. (**B**), Nematicidal activity of *Streptomyces* sp. AE170020 broth between pH 1 and 11, M9 buffer was served as control; (**C**), Nematicidal activity of the supernatant of *Streptomyces* sp. AE170020 with or without treatment with protease K. Control: supernatant of GSS medium. Data are shown as the mean ± SD of two independent experiments with three replicates.

As shown in Fig. 2A, the 3-days to 10-days fermentation supernatant of strain AE170020 had evident activity against *B. xylophilus*, with mortality ranging from 62.8 ± 2.5% to 94.2 ± 0.8%. The highest mortality was observed when *B. xylophilus* was exposure to 5-days and 6-days fermentation broths. The broth of GSS medium led to a modest mortality rate of 6.7 ± 0.6%.

The nematicidal substances of strain AE170020 were also analyzed for their stability at different pH values. It was found that the supernatant of strain AE170020 exhibited high nematicidal activity within a pH range of 4.0–11.0. However, the nematicidal activity slightly decreased at pH 4.0 and 5.0, and no mortality was observed in strong acidic environments (pH 1.0–3.0) (Fig. 2B).

The supernatant of strain AE170020 was also analyzed for its stability towards protease digestion. After hydrolysis with protease K, the supernatant of strain AE170020 also showed high nematicidal activity with a mortality of 91.6 ± 2.9% against *B. xylophilus* (Fig. 2C).

Therefore, we speculated that strain AE170020 produces some extracellular substances to kill the nematode and the nematicidal substances of the fermentation broth are not protein but could be secondary metabolites instead.

### Nematicidal activity of strain AE170020 extracts

**Figure 3:**
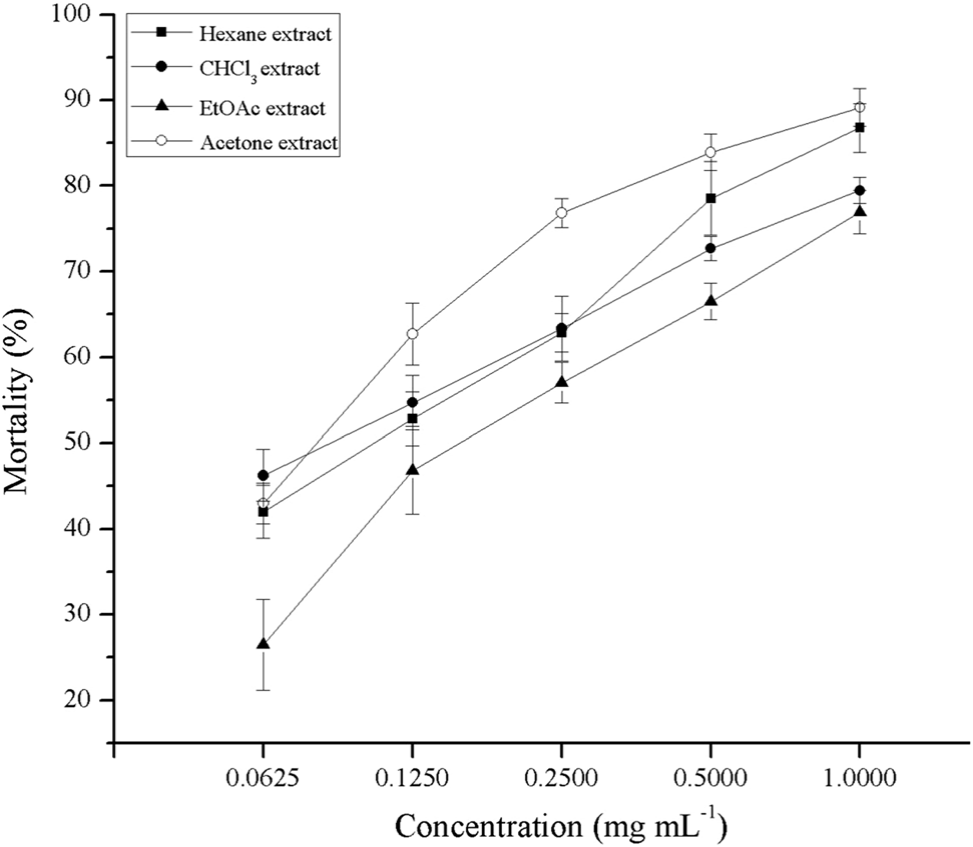
Nematicidal activity of solvent-partitioned extracts of *Streptomyces* sp. AE170020 against *B. xylophilus* after 24 h treatment. All the data are shown as the mean ± SD (n = 9).

The mortality rates of different solvent partitions were assessed as shown in Fig. 3. The extracts of different solvents clearly display a concentration-dependent nematicidal activity against *B. xylophilus*. The phase extracted by acetone showed the highest nematicidal activity with 89.2 ± 2.2% mortality at a concentration of 1.0 mg mL^−1^, followed by the hexane extract. The extracts of CHCl_3_ and EtOAc also exhibited nematicidal activities relative to the control group with a mortality rate of 5.7 ± 0.7% (Fig. 3).

### Purification and identification of bioactive compounds

**Figure 4:**
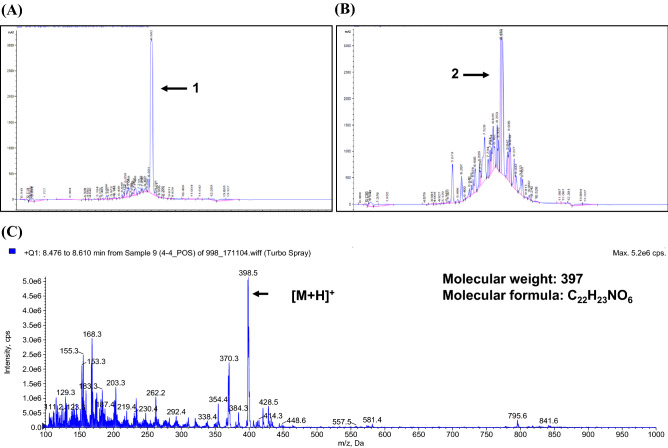
(**A**) HPLC profile of alloaureothin (**B**) HPLC profile of aureothin (**C**) LC/MS profile of alloaureothin and aureothin.

**1** was obtained as a yellow amorphous solid and showed UV spectrum (l max, 255, 334 nm). **2** was obtained as a yellow Prism and exhibited the same UV spectrum as **1** (l max, 255, 334 nm). Its physico-chemical properties are summarized in Table 2. As a result of analyzing the molecular weights of the two compounds by LC/MS, it was confirmed that they were the same molecular weight. The molecular formula was established as C_22_H_23_NO_6_ from HR-ESI–MS data (m/z 398.1628). The purified **1** and **2** were a yellow powder; ESI–MS [M+H]^+^ m/z 398.0 (calcd for C_22_H_23_NO_6_, 397.0) (Fig. 4C).

**Figure 5:**
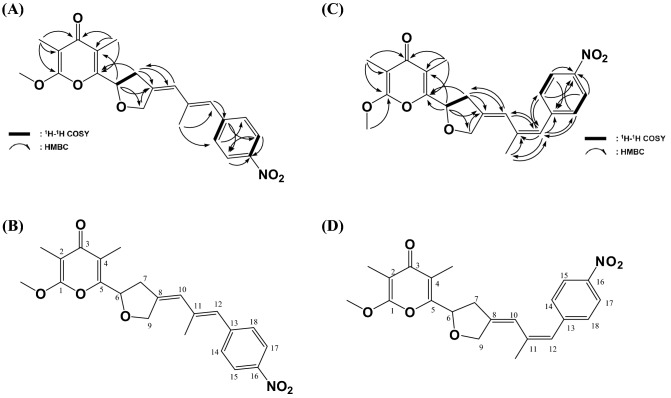
Chemical structure of (**A**,**B**) alloaureothin and (**C**,**D**) aureothin.

^1^H-NMR and ^13^C-NMR of the **1** revealed that 22 carbons and 23 protons were present. ^1^H-NMR spectra showed an methoxyl proton at 3.97, 2.07, 1.97, 1.81 ppm (Fig. S1A). The ^13^C-NMR spectra showed that there were six aromatic carbon atoms between 146.2 ppm and 120.7 ppm and four olefin carbon atoms at 147.4, 139.8, 128.0, 120.7 ppm, and one ketone carbon was observed at 182.8 ppm (Fig. S1C). The cross peak was analyzed by measuring the HMQC spectrum to confirm the presence of quarternary carbon and the link between the protons and carbon. As a result, all proton-bearing carbon (^1^J_C-H_) could be identified and assigned to complete the ^1^H-^13^C NMR chart (Table 1). H-6 (δ_H_ 5.23) and C-6 (δ_C_ 75.0), H-7 (δ_H_ 2.95, 3.05) and C-7 (δ_C_ 38.5), H-9 (δ_H_ 4.58, 4.45) and C-9 (δ_C_ 71.3), H-10 (δ_H_ 6.40) and C-10 (δ_C_ 120.9), H-12 (δ_H_ 6.46) and C-12 (δ_C_ 128.0), H-14 (δ_H_ 7.49) and C-14 (δ_C_ 130.8), H-15 (δ_H_ 8.15) and C-15 (δ_C_ 124.6), H-17 (δ_H_ 8.15) and C-17 (δ_C_ 124.6) and H-18 (δ_H_ 7.49) and C-18 (δ_C_ 130.8), 1-O-Me (δ_H_ 3.97) and 1-O-Me (δ_C_ 56.5), 2-Me (δ_H_ 1.81) and 2-Me (δ_C_ 7.1), 4-Me (δ_H_ 1.97)와 4-Me (δ_C_ 9.4), 11-Me (δ_H_ 2.07) and 11-Me (δ_C_ 24.3) was confirmed (Fig. S2A). In addition, the cross peak between ^1^H-^1^H was confirmed through the ^1^H-^1^H COSY spectrum, and the HMBC spectrum was measured to confirm the position of carbon in the 2, 3 and 4 bonds from the proton (Fig. S2B,C). As a result, it was confirmed that this is an alloaureothin structure (Fig. 5A,B). The physicochemical properties of alloaureothin are shown in Table 2.

**Table 1:** ^1^H (400 MHz) and ^13^C (100 MHz) NMR data of compound **1** and **2** in CDCl_3_.

Position	**1**		**2**	
	δ_H_	δ_C_	δ_H_	δ_C_
1	–	164.5, s	–	162.1, s
2	–	100.7, s	–	100.1, s
3	–	182.8, s	–	180.6, s
4	–	120.7, s	–	120.2, s
5	–	157.7, s	–	154.6, s
6	5.23 (1H, t, *J* = 6.9 Hz)	75.0, d	5.15 (1H, t, *J* = 7.0 Hz)	73.3, d
7	2.95 (1H, dd, *J* = 6.4, 16.6 Hz)	38.5, t	2.97 (1H, dd, *J* = 6.4, 16.0 Hz)	38.3, t
	3.05 (1H, dd, *J* = 7.3, 16.4 Hz)		3.07 (1H, dd, *J* = 6.3, 16.1 Hz)	
8	–	143.5, s	–	144.2, s
9	4.58 (1H, d,* J* = 14.5 Hz)	71.3, t	4.90 (1H, d, *J* = 14.2 Hz)	70.1, t
	4.45 (1H, d, *J* = 15.0 Hz)		4.75 (1H, d, *J* = 14.1 Hz)	
10	6.40 (1H , br, s)	120.9, d	6.21 (1H, br, s)	126.0, d
11	–	139.8, s	–	138.5, s
12	6.46 (1H , br, s)	128.0, d	6.38 (1H, br, s)	128.4, d
13	–	146.2, s	–	140.6, s
14, 18	7.49 (2H, d, *J* = 8.6 Hz)	130.8, d	7.40 (2H, d, *J* = 8.7 Hz)	129.6, d
15, 17	8.15 (2H, d, *J* = 8.7 Hz)	124.6, d	8.21 (2H, d, *J* = 8.8 Hz)	123.6, d
16	–	147.4, s	–	146.1, s
1-O-Me	3.97 (3H, s)	56.5, q	3.96 (3H, s)	55.3, q
2-Me	1.81 (3H, s)	7.1, q	1.86 (3H, s)	6.9, q
4-Me	1.97 (3H, s)	9.4, q	2.04 (3H, s)	9.5, q
11-Me	2.07 (3H, s)	24.3, q	2.06 (3H, s)	17.7, q

**Table 2:** Physicochemical properties of alloaureothin and aureothin.

Physico-chemical properties	Alloaureothin	Aureothin
Appearance	Yellow amorphous solid	Yellow prism
Molecular formula	C_22_H_23_NO_6_	C_22_H_23_NO_6_
found	398.4 [M+H^+^]	398.4 [M+H^+^]
calcd	397.4	397.4

The **2** was ^1^H-NMR spectra showed an methoxyl proton at 3.95, 2.06, 2.04, 1.86 ppm (Fig. S1B). The ^13^C-NMR spectra showed that there were six aromatic carbon atoms between 146.2 ppm and 120.7 ppm and four olefin carbon atoms at 146.1, 138.5, 128.4, 120.2 ppm, and one ketone carbon was observed at 180.6 ppm (Fig. S1D). The cross peak was analyzed by measuring the HMQC spectrum to confirm the presence of quarternary carbon and the link between the protons and carbon. As a result, all proton-bearing carbon (^1^J_C-H_) could be identified and assigned to complete the ^1^H-^13^C NMR chart (Table 1). H-6 (δ_H_ 5.15) and C-6 (δ_C_ 73.3), H-7 (δ_H_ 3.07, 2.97) and C-7 (δ_C_ 38.3), H-9 (δ_H_ 4.90, 4.75) and C-9 (δ_C_ 70.1), H-10 (δ_H_ 6.21) and C-10 (δ_C_ 126.0), H-12 (δ_H_ 6.38) and C-12 (δ_C_ 128.4), H-14 (δ_H_ 7.40) and C-14 (δ_C_ 129.6), H-15 (δ_H_ 8.21) and C-15 (δ_C_ 123.6), H-17 (δ_H_ 8.21) and C-17 (δ_C_ 123.6) and H-18 (δ_H_ 7.40) and C-18 (δ_C_ 129.6), 1-O-Me (δ_H_ 3.96) and 1-O-Me (δ_C_ 55.3), 2-Me (δ_H_ 1.86) and 2-Me (δ_C_ 6.9), 4-Me (δ_H_ 2.04) and 4-Me (δ_C_ 9.5), 11-Me (δ_H_ 2.06) and 11-Me (δ_C_ 17.7) was confirmed (Fig. S2D). In addition, the cross peak between ^1^H-^1^H was confirmed through the ^1^H-^1^H COSY spectrum, and the HMBC spectrum was measured to confirm the position of carbon in the 2, 3 and 4 bonds from the proton (Fig. S2E,F). As a result, it was confirmed that this is an aureothin structure (Fig. 5C,D). The physicochemical properties of aureothin are shown in Table 2.

Two compounds isolated from *Streptomyces* sp. AE170020, aureothin and alloaureothin, were confirmed to be isomers around a double bond. It was confirmed that both compounds should be stored below 0 °C and stored under dark conditions because they are weak to light.

### Effects of alloaureothin and aureothin on different developmental stages of *B. xylophilus*

**Figure 6:**
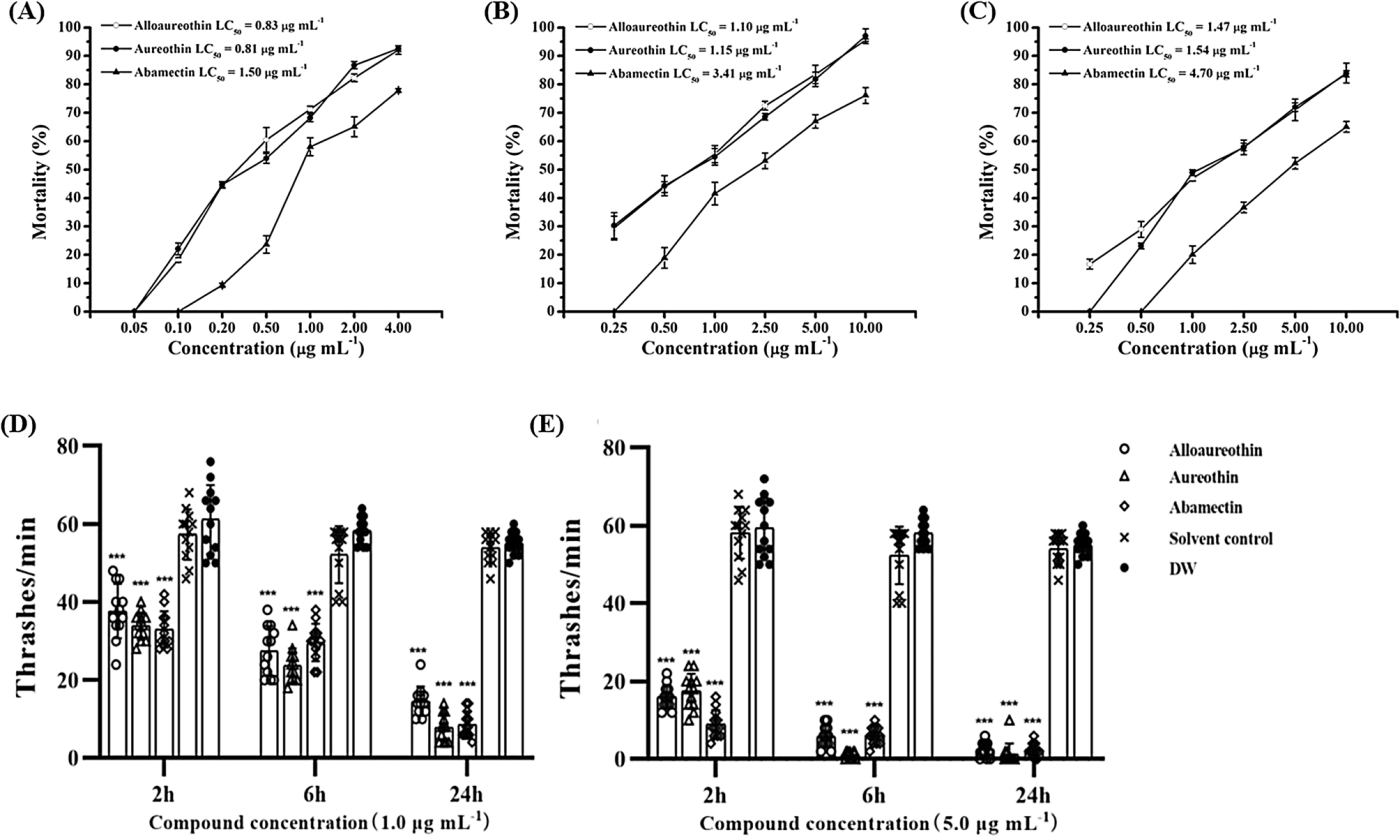
Nematicidal activity of alloaureothin and aureothin on different life stages of *Bursaphelenchus xylophilus* after 24 h. (**A**), J2 stage; (**B**), J3 stage, and (**C**), J4 stage/adults. Abamectin served as the positive control and no mortality was observed when nematodes were treated with 2 μL DMSO. Data were obtained from three experiments with three replicates each and are shown as the mean ± SD. Influences of alloaureothin, aureothin and abamectin on the locomotor activity of *B. xylophilus*. Nematodes were exposure to the compound at the concentration of (**D**); 1.0 μg mL^−1^, (**E**); 5.0 μg mL^−1^, respectively. The graphs show the mean ± SD of two trials with six repetitions.

Alloaureothin and aureothin showed highly significant activity against J2s, J3s, and J4s/adults of *B. xylophilus*, and as expected, the two compounds concentration-dependently killed *B. xylophilus*. Among these, the J2s stage is the most sensitive to the two compounds, followed by J3s and J4s/adults stages, and this sensitivity pattern is similar to that observed with the positive control, abamectin. After 24-h exposure, the LC_50_ values of alloaureothin on different life stages of *B. xylophilus*: [J2s (Fig. 6A), J3s (Fig. 6B), and J4s/adults (Fig. 6C)] were 0.83, 1.10, and 1.47 μg mL^−1^, respectively, and the values of aureothin were 0.81, 1.15, and 1.54 μg mL^−1^ respectively. Compared with abamectin, both compounds showed higher nematicidal activity against *B. xylophilus* at all tested life stages, and the two compounds showed similar mortality rates against *B. xylophilus*.

### Alloaureothin and aureothin suppressed the thrashing potential of nematode

After being treated with alloaureothin and aureothin, nematode thrashing frequencies were decreased, and this decrease was time-dependent and concentration-dependent. Nematode thrashing frequencies were significantly reduced by alloaureothin (14 ± 4), aureothin (8 ± 3), and abamectin (9 ± 3) at a concentration of 1 μg mL^−1^ as compared with the solvent-treated group (54 ± 4) and non-treated control (55 ± 3) after 24 h (Fig. 6D). Upon exposure to the active compounds at a concentration of 5.0 μg mL^−1^, nematodes almost had no movement after 24-h treatment (Fig. 6E).

### Effects of alloaureothin and aureothin on the reproductive traits of *B. xylophilus*

**Figure 7:**
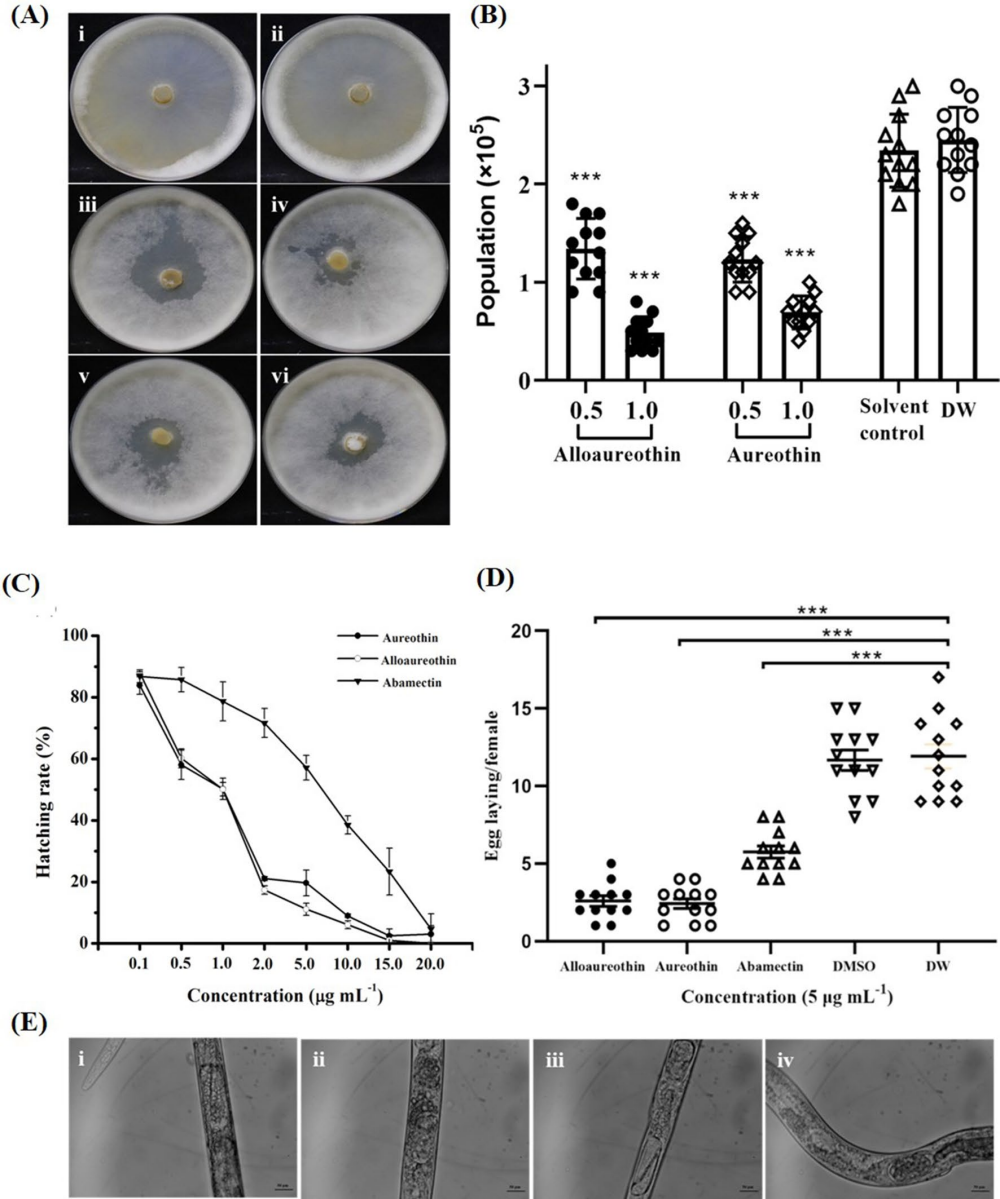
Alloaureothin and aureothin disrupted reproductive traits in *B. xylophilus*. *B. cinerea* in Petri dishes after inoculation with *B. xylophilus* untreated (**Ai**) or treated with DMSO (**Aii**), alloaureothin (0.5 μg mL^−1^) (**Aiii**), alloaureothin (1.0 μg mL^−1^) (**Aiv**), aureothin (0.5 μg mL^−1^) (**Av**), aureothin (1.0 μg mL^−1^) (**Avi**), and the number of nematodes per dish in each treatment were also calculated (**B**). The results were recored after 7 days inoculation. Values are mean ± SD of six replicates from two trials. Effects of alloaureothin and aureothin on egg hatching and egg deposition of nematode. (**C**) Effects on hatchability after 48 h; (**D**) Eggs laid by per female nematodes after 24 h; (**E**) Representative images of female nematodes showing eggs when exposure to different compounds [(i) alloaureothin, (ii) aureothin, (iii) abamectin, (iv) DMSO, (vi) none]. Values in graph A are mean ± SD of three replicates from two trials, whereas graph B shows the mean ± SD of two experiments with six repetitions.

The mycelia of *B. cinerea* were almost completely consumed by nematodes after 7 days in the non-treated plates and the DMSO-treated group exhibited a similar clearance zone to the non-treated plates (Fig. 7Ai,ii). In contrast, in the plates treated with two active compounds, clearance zones were clearly reduced (Fig. 7Aiii–vi). Alloaureothin and aureothin were found to decrease the mean nematode numbers to 1.3 × 10^5^ ± 0.5, 1.2 × 10^5^ ± 0.7 at the concentration of 0.5 μg mL^−1^, while the values were 0.3 × 10^5^ ± 0.1 and 0.2 × 10^5^ ± 0.1 at the concentration of 1 μg mL^−1^ compared with non-treated controls (2.5 × 10^5^ ± 0.4) (Fig. 7B).

### Alloaureothin and aureothin affected the hatching and fecundity of nematode

The effect of active compounds on reproductive activity was measured in vitro by direct contact. The results suggested that the hatching rate of *B. xylophilus* was significantly inhibited by the test compounds in a dose-dependent manner (Fig. 7C). Non-treated and solvent controls presented a hatching rate of 92.1 ± 2.1% and 87.5 ± 3.3% respectively, whereas alloaureothin and aureothin at a concentration of 20 μg mL^−1^ treated nematode eggs almost failed to hatch. In addition, both compounds exhibited a significantly lower hatching rate compared with abamectin.

Furthermore, two active compounds also adversely influenced the fecundity of gravid adults at the concentration of 5 μg mL^−1^. Eggs laid by a single female in non-treatment and DMSO-treated were 12 ± 3 and 12 ± 2, respectively (Fig. 7D). However, the values were reduced to 3 ± 1, 2 ± 1, and 6 ± 1 by alloaureothin, aureothin, and abamectin. Microscopic imaging also revealed the inability of female nematodes to lay eggs, leading to the accumulation of eggs inside nematodes (Fig. 7E).

### Suppression of pine wilt disease under pot condition

**Figure 8:**
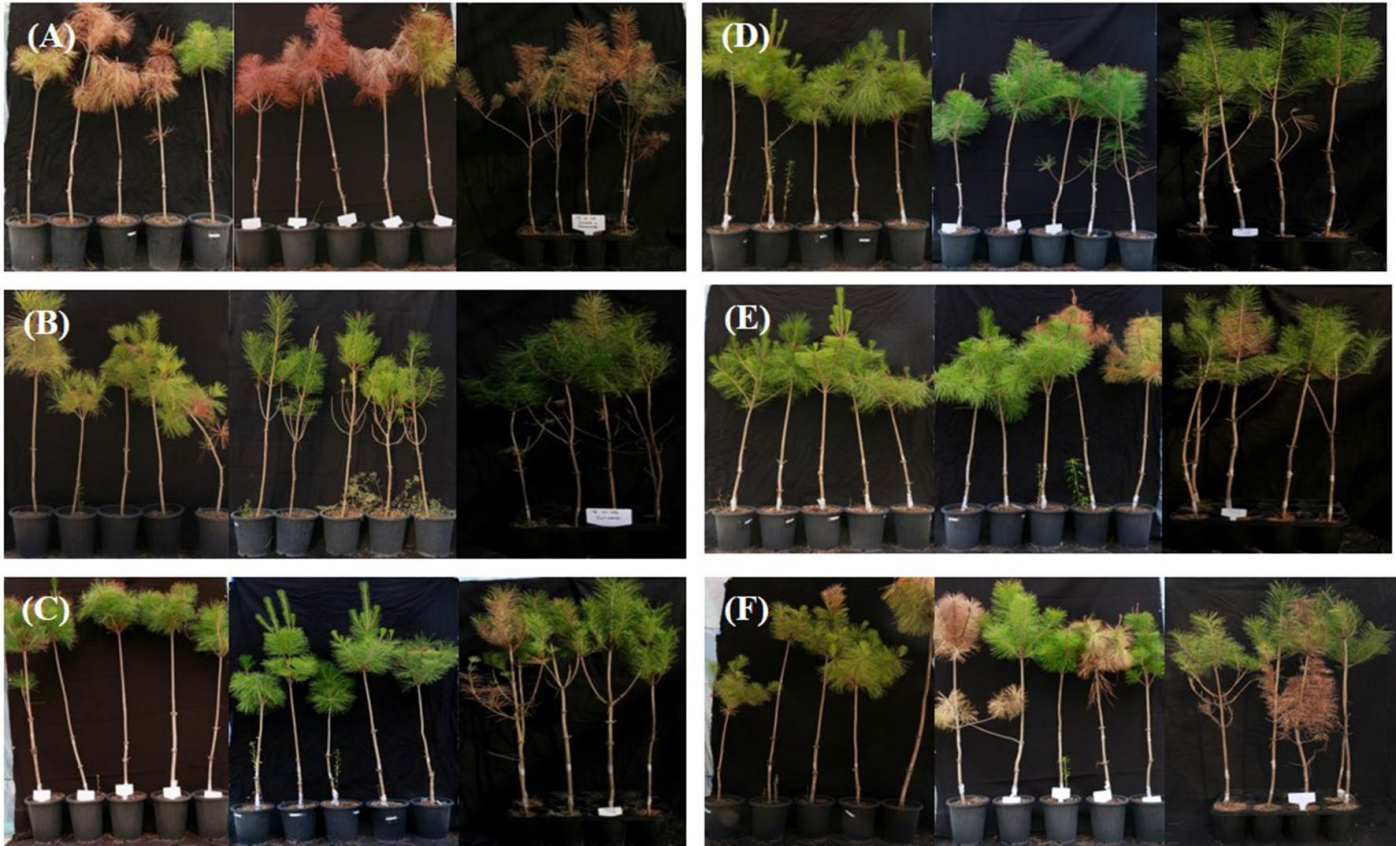
Effects of *Streptomyces* sp. AE170020 mycelium extracts from the first pot test on *P. thunbergii* caused by *B. xylophilus* on PWD. (**A**), negative control; (**B**), non-inoculation; (**C**), abamectin-treatment. (**D**–**F**), extracts of strain 680,998 at the dose of 7.2, 3.6 and 1.8 mg per seedling, respectively.

Successful nematode infection in pine trees is visualized by the presence of needle discoloration, and these visual symptoms were registered to understand and compare the effect of compounds on tree survival. As shown in Table 3, after inoculation with *B. xylophilus*, 90% of *P. densiflora* plants in the control group died after 60 days. The injection of acetone extracts of *Streptomyces* sp. AE170020 and abamectin into pine trees at test concentrations successfully suppressed the development of PWD in pine trees artificially infected with *B. xylophilus*. Remarkably, extracts of strain AE170020 exhibited 100% control efficacy at a concentration of 7.2 mg per tree (Fig. 8).

**Table 3:** Control efficacy of pine wilt disease in 4-year-old *Pinus desiflora* treated with acetone extarct of strain AE170020 under pot conditions and nematode population per gram of stem fresh weight recovered from pine trees.

Treated sample	Concentration (mg per seedling)	Estimated number of nematodes^1^
Acetone extract of strain AE170020	7.2	350 ± 53^a^
	3.6	387 ± 84^a^
	1.8	720 ± 107^c^
Abamectin	3.6	433 ± 184^a^
Untreated	**–**	3426 ± 576^b^

^1^Nematode population was measured 60 days after inoculation. Values represent the mean ± SD. Means in the same column followed by the same letters are not significantly different.

Moreover, after 60 days, the nematode population was four to nine times larger in the nematode control group than in the compound-injection groups, with numbers increasing from about 2000 nematodes at inoculation to 3426 ± 576 nematodes per gram of plant tissue (Table 3). The lowest density of nematodes (350 ± 84) was recovered from pine trees treated with strain extract at the concentration of 7.2 mg per tree.

### Variation of chemical traits after inoculation

**Figure 9:**
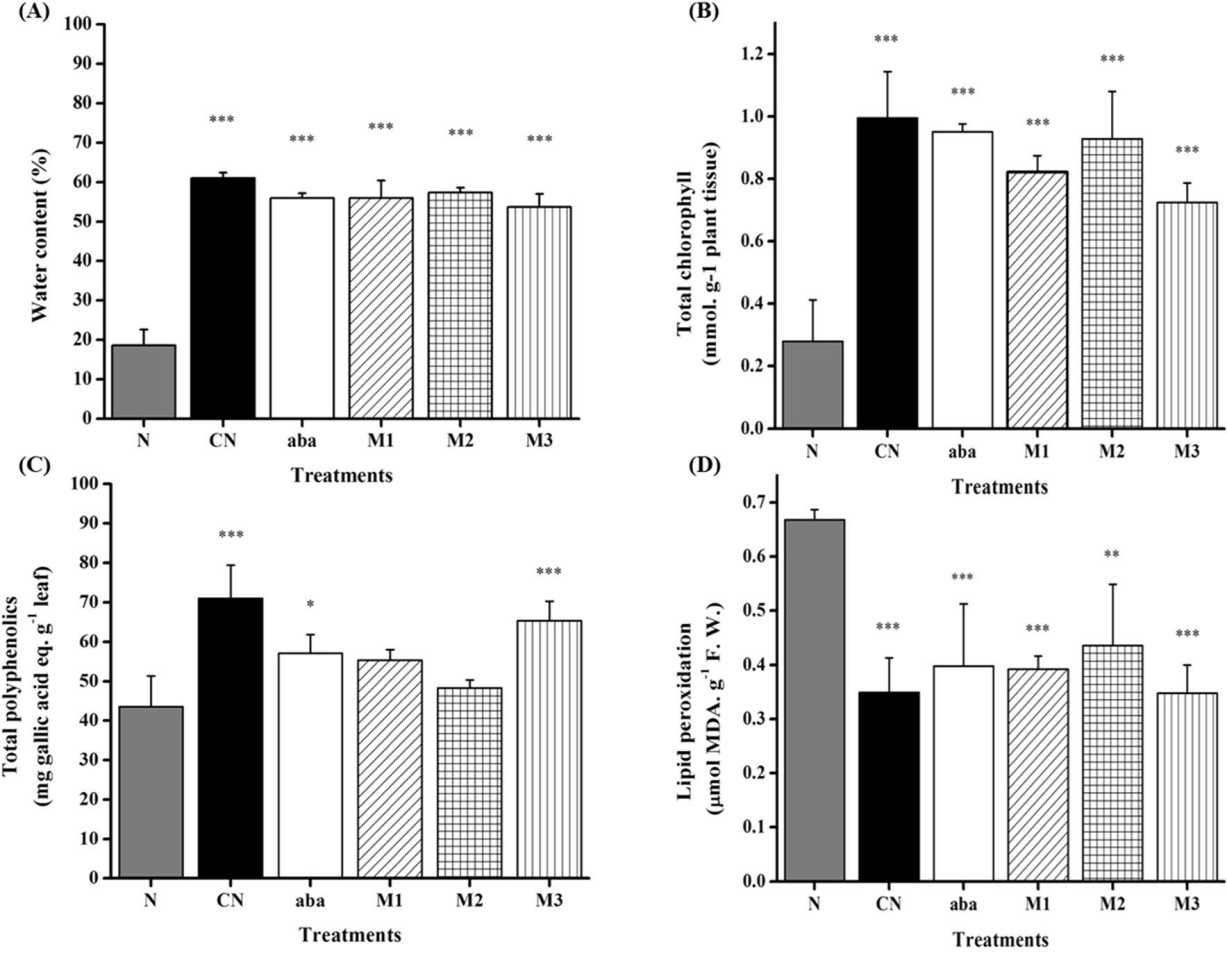
Effect of compound treatments on (**A**) total chlorophyll content, (**B**) total polyphenolics content, (**C**) lipid peroxidation, (**D**) water content in *Pinus densiflora* seedlings during nematode infection. N, CN, aba, M1, M2 and M3 represent the same treatments as in the pot test. All the data are shown as the mean ± SD. N, negative control; CN, solvent-treatment; aba, abamectin; M1, M2 and M3 represent the acetone extract at the concentration of 7.2, 3.6 and 1.8 mg per seedling, respectively. All the data were shown as the mean ± SD.

It was clearly seen that injection of abamectin and acetone extract of strain AE170020 successfully prevented the reductions of water content (Fig. 9A), total chlorophyll content (Fig. 9B), and total polyphenolics (Fig. 9C). The increasing concentration of malondialdehyde (MDA) in the nematode-inoculation control group suggested a high degree of lipid oxidative damage caused by PWNs (Fig. 9D), and abamectin and strain extracts protected pine trees from this damage.

## Discussion

PWD, which is caused by PWN, is one of the most destructive diseases in trees of the genus *Pinus* and is responsible for environmental and economic losses around the world. The frequently used nematicides to control the disease are chemical nematicides. However, these chemical nematicides reportedly have deleterious effects on human health and the environment. Biological control of the PWD using microorganisms can be a safe, cost-effective, and efficient method. Endophytes reside with plant tissues without causing any signs of disease or infections and are rich sources of bioactive substances and promising biocontrol agents because of the direct effects of metabolites to induce mortality in nematodes. Many endophytic bacteria are reported to exert nematicidal activity by producing nematicidal toxins^26–28^.

In the present study, an endophytic bacteria, *Streptomyces* sp. AE170020, isolated from pine trees was able to effected *B. xylophilus*. We further investigated the active substances of this strain and hypothesized that some secondary metabolites may be responsible for the nematicidal activity. Subsequently, the nematicidal substances were purified by using bioassay-guided fractionation and two active compounds, alloaureothin and aureothin, were identified. Alloaureothin and aureothin, which are polypropionates with a nitro group belonging to the class of polyketides, exhibited high activity against *B. xylophilus*. Two potent compounds were previously isolated from several actinomycetes and displayed interesting biological activities. Aureothin isolated from the mycelium of *Streptomyces thioluteus*^29^ and *Streptomyces* sp. MM23^27^ was reported to exhibit anti-*Helicobacter*, antitumor, and antifungal activities^27,30^. In accordance with our results, previous studies have also shown that aureothin exerts nematicidal activity^29^. Alloaureothin obtained from *Streptomyces* sp. MM23 was reported to exhibit growth inhibitory effects against human fibrosarcoma HT1080 cells with an IC_50_ value of 30 μM^27^. In our present study, alloaureothin also showed high nematicidal activity against *B. xylophilus*.

It is important to investigate the mode of action of active compounds in the practice of nematode control, because it can provide useful information for avoiding resistance and choosing appropriate way of delivery. Further research was conducted to evaluate the effects of two potent compounds, alloaureothin and aureothin, on the growth, reproduction, and development of nematodes. The present results showed that both compounds were lethal to all developmental stages of nematode and displayed a lower LC_50_ value than the commercial nematicide, abamectin. In addition, the nematode population was significantly reduced by two active compounds, which supports that both compounds control the reproductive behavior of nematodes. Eggs hatchability is one of the most important parameters that affects population numbers and it has been suggested that chemicals with high embryonic lethality can be potential nematicides^30,31^. The two compounds obtained in our research both effectively inhibited the hatchability of nematode eggs and can be considered as potential nematicides.

The trunk injection method involves injection of pesticides directly into the tree trunk; the liquids are then transported through the plant’s conductive tissues to the site of action. This method has been successfully used in biocontrol of PWD^32,33^. Abamectin, a member of macrocyclic lactones, has been widely used in biocontrol of various kinds of parasitic nematodes, and several studies have shown its ability in controlling PWD^34,35^. In our current study, in vivo experiments under greenhouse conditions using the trunk injection method showed that the extracts of *Streptomyces* sp. AE170020 and abamectin successfully suppressed the development of PWD. To monitor the multiplication of nematodes in plants with or without the active substances, pine trees in each treatment were harvested and the number of nematodes was calculated. The compounds did not kill all the nematodes but significantly reduced the total number of nematodes compared with the nematode-control group (3426 ± 576 nematode per gram stem). These results indicate that these compounds prevented, or at least slowed, the beginning and progression of PWD. Although the two compounds isolated from different *Streptomyces* sp. than used herein have already been described and their biological activities have been studied, to our best knowledge, this is the first report exploring the nematicidal activity of alloaureothin and applying strain *Streptomyces* sp. AE170020 to the biocontrol of PWD under pot conditions.

In addition to the external symptoms, the examination of other characteristics of pine trees after treatments allowed a better assessment of the physiological impact of nematode infection and compound injection. Specific water content was used as a proxy for water stress, which is usually associated with PWD. A decrease in chlorophyll content was previously described in PWN-inoculated pine seedlings and is regarded as a symptom of the advanced stage of the PWD, which is induced by water deficiency in leaves^36^. Phenolic substances have been considered responsible for the browning of injured or pathogen-infected plant tissues, and PWD may be a result of the production of such compounds^37^. In the current study, acetone extract of strain *Streptomyces* sp. AE170020 prevented the reductions of water content, total chlorophyll, and total polyphenolics. Lipid peroxidation is an indicator of membrane cellular damage and usually occurs in plants after PWN inoculation as a result of xylem parenchyma cell necrosis and partial destruction of the cortex and phloem^38^. The increasing concentration of malondiadehyde in the nematode-control group suggested a high degree of lipid oxidative damage caused by PWN, and the active substances successfully protected pine trees from this damage.

Therefore, this study explored an endophytic strain, *Streptomyces* sp. AE170020, and its related compounds for defense against *B. xylophilus *in vitro and in vivo under greenhouse conditions. These results support the development of endophytic microorganisms as alternatives for the management of PWD. Further investigation is needed to understand the molecular mechanisms responsible for the nematicidal activity. In addition, scale-up, modification for this strain, safety evaluation, and risk assessment should be further developed to control *B. xylophilus* in actual agricultural applications.

## Supplementary Information

Supplementary Figures.

## References

[1] Kikuchi T, Cotton JA, Dalzell JJ, Hasegawa K, Kanzaki N, McVeigh P, Takanashi T, Tsai IJ, Assefa SA, Cock PJ (2011). Genomic insights into the origin of parasitism in the emerging plant pathogen *Bursaphelenchus xylophilus*. PLoS Pathog..

[2] Mamiya Y (1988). History of pine wilt disease in Japan. J. Nematol..

[3] Cheng XY, Cheng FX, Xu RM, Xie BY (2008). Genetic variation in the invasive process of *Bursaphelenchus xylophilus* (Aphelenchida: Aphelenchoididae) and its possible spread routes in China. Heredity.

[4] Mota MM, Braasch H, Bravo MA, Penas AC, Burgermeister W, Metge K, Sousa E (1999). First report of *Bursaphelenchus xylophilus* in Portugal and in Europe. Nematology.

[5] Yamada, T. Biochemical responses in pine trees affected by pine wilt disease. In *Pine Wilt Disease* 223–234 (Springer, 2008).

[6] Gowen, S. Chemical control of nematodes: efficiency and side-effects. *FAO Plant Production and Protection Paper (FAO)*. (1997).

[7] Tian BY, Yang JK, Zhang KQ (2007). Bacteria used in the biological control of plant-parasitic nematodes: Populations, mechanisms of action, and future prospects. FEMS Microbiol. Ecol..

[8] Yu J, Du G, Li RG, Li L, Li Z, Zhou CJ, Chen CC, Guo DS (2015). Nematicidal activities of bacterial volatiles and components from two marine bacteria, *Pseudoalteromonas marina* strain H-42 and *Vibrio atlanticus* strain S-16, against the pine wood nematode, *Bursaphelenchus xylophilus*. Nematology.

[9] Kerry, B. Biological control of nematodes: prospects and opportunities. Plant Nematode Problems and their Control in the Near East Region 79–92 (1997).

[10] Li J, Zou CG, Xu JP, Ji XL, Niu XM, Yang JK, Huang XW, Zhang KQ (2015). Molecular mechanisms of nematode-nematophagous microbe interactions: Basis for biological control of plant-parasitic nematodes. Annu. Rev. Phytopathol..

[11] Tranier MS, Pognat-Gros J, Quiroz RDLC, González CNA, Mateille T, Roussos S (2014). Commercial biological control agents targeted against plant-parasitic root-knot nematodes. Braz. Arch. Biol. Technol..

[12] Hallmann J, Quadt-Hallmann A, Mahaffee WF, Kloepper JW (1997). Bacterial endophytes in agricultural crops. Can. J. Microbiol..

[13] Strobel GA (2003). Endophytes as sources of bioactive products. Microbes Infect..

[14] Strobel G, Daisy B, Castillo U, Harper J (2004). Natural products from endophytic microorganisms. J. Nat. Prod..

[15] Zheng LJ, Li GH, Wang XB, Pan WZ, Li L, Hua L, Liu FF, Dang LZ, Mo MH, Zhang KQ (2008). Nematicidal endophytic bacteria obtained from plants. Ann. Microbiol..

[16] Strzelczyk E, Li CY (2000). Bacterial endobionts in the big non-mycorrhizal roots of Scots pine (*Pinus sylvestris* L.). Microbiol. Res..

[17] Pirttilä AM, Laukkanen H, Pospiech H, Myllylä R, Hohtola A (2000). Detection of intracellular bacteria in the buds of Scotch pine (*Pinus sylvestris* L.) by in situ hybridization. Appl. Environ. Microbiol..

[18] Bal A, Anand R, Berge O, Chanway CP (2012). Isolation and identification of diazotrophic bacteria from internal tissues of *Pinus contorta* and *Thuja plicata*. Can. J. For. Res..

[19] Carrell AA, Frank AC (2014). *Pinus flexilis* and *Picea engelmannii* share a simple and consistent needle endophyte microbiota with a potential role in nitrogen fixation. Front. Microbiol..

[20] Ponpandian LN, Rim SO, Shanmugam G, Jeon J, Park YH, Lee SK, Bae H (2019). Phylogenetic characterization of bacterial endophytes from four Pinus species and their nematicidal activity against the pine wood nematode. Sci. Rep..

[21] Park IK, Kim J, Lee SG, Shin SC (2007). Nematicidal activity of plant essential oils and components from ajowan (*Trachyspermum ammi*), allspice (*Pimenta dioica*) and litsea (*Litsea cubeba*) essential oils against pine wood nematode (*Bursaphelenchus xylophilus*). J. Nematol..

[22] Gao H, Qi G, Yin R, Zhang H, Li C, Zhao X (2016). *Bacillus cereus* strain S2 shows high nematicidal activity against *Meloidogyne incognita* by producing sphingosine. Sci. Rep..

[23] Cheng L, Xu S, Xu C, Lu H, Zhang ZQ, Zhang DX, Mu W, Liu F (2017). Effects of trans-2-hexenal on reproduction, growth and behaviour and efficacy against the pinewood nematode, *Bursaphelenchus xylophilus*. Pest. Manag. Sci..

[24] Liu MJ, Hwang BS, Jin CZ, Li WJ, Park DJ, Seo ST, Kim CJ (2019). Screening, isolation and evaluation of a nematicidal compound from actinomycetes against the pine wood nematode, *Bursaphelenchus xylophilus*. Pest. Manag. Sci..

[25] Nunes da Silva M, Solla A, Sampedro L, Zas R, Vasconcelos MW (2015). Susceptibility to the pinewood nematode (PWN) of four pine species involved in potential range expansion across Europe. Tree Physiol..

[26] Ainsworth EA, Gillespie KM (2007). Estimation of total phenolic content and other oxidation substrates in plant tissues using Folin-Ciocalteu reagent. Nat. Protoc..

[27] Ueda JY, Hashimoto J, Nagai A, Nakashima T, Komaki H, Anzai K, Harayama S, Doi T, Takahashi T, Nagasawa K (2007). New aureothin derivative, alloaureothin, from *Streptomyces* sp. MM23. J. Antibiot..

[28] Mekete T, Hallmann J, Kiewnick S, Sikora R (2009). Endophytic bacteria from Ethiopian coffee plants and their potential to antagonise Meloidogyne incognita. Nematology.

[29] Hirata Y, Nakata H, Yamada K, Okuhara K, Naito T (1961). The structure of aureothin, a nitro compound obtained from *Streptomyces thioluteus*. Tetrahedron.

[30] Taniguchi M, Watanabe M, Nagai K, Suzumura K, Suzuki K, Tanaka A (2000). γ-Pyrone compounds with selective and potent anti-*Helicobacter pylori* activity. J. Antibiot..

[31] Rajasekharan SK, Lee JH, Ravichandran V, Lee J (2017). Assessments of iodoindoles and abamectin as inducers of methuosis in pinewood nematode, *Bursaphelenchus xylophilus*. Sci. Rep..

[32] Takai K, Soejima T, Suzuki T, Kawazu K (2000). Emamectin benzoate as a candidate for a trunk-injection agent against the pine wood nematode, *Bursaphelenchus xylophilus*. Pest Manag. Sci. Formerly Pesticide Sci..

[33] Takai K, Suzuki T, Kawazu K (2003). Development and preventative effect against pine wilt disease of a novel liquid formulation of emamectin benzoate. Pest Manag. Sci. Formerly Pesticide Sci..

[34] Kwon HR, Choi GJ, Choi YH, Jang KS, Sung ND, Kang MS, Moon Y, Lee SK, Kim JC (2010). Suppression of pine wilt disease by an antibacterial agent, oxolinic acid. Pest. Manag. Sci Formerly Pesticide Sci..

[35] James R, Tisserat N, Todd T (2006). Prevention of pine wilt of scots pine (*Pinus sylvestris*) with systemic abamectin injections. Arboric. Urban For..

[36] Chen Y, Ye J, Wei C, Pan H (2005). Effects of pine wood nematode (PWN) infection on water regime and metabolism of related to hosts. Acta Phytopathol. Sinica.

[37] Futai K (2003). Abnormal metabolites in pine wood nematode-inoculated Japanese black pine. Nematol. Res..

[38] Yamada, T. Biochemical responses in pine trees affected by pine wilt disease. In *Pine Wilt Disease*, 223–234 10.1007/978-4-431-75655-2_22 (Springer, 2008).

