# Supplementary material for: New discovery on the nematode activity of aureothin and alloaureothin isolated from endophytic bacteria Streptomyces sp. AE170020
Source: Sci Rep. 2022 Mar 10;12:3947. doi: 10.1038/s41598-022-07879-w (PMC8913828; doi:10.1038/s41598-022-07879-w)
Supplement: Supplementary file 1 — Supplementary Figures. [file 41598_2022_7879_MOESM1_ESM.pptx]

## Slide 1
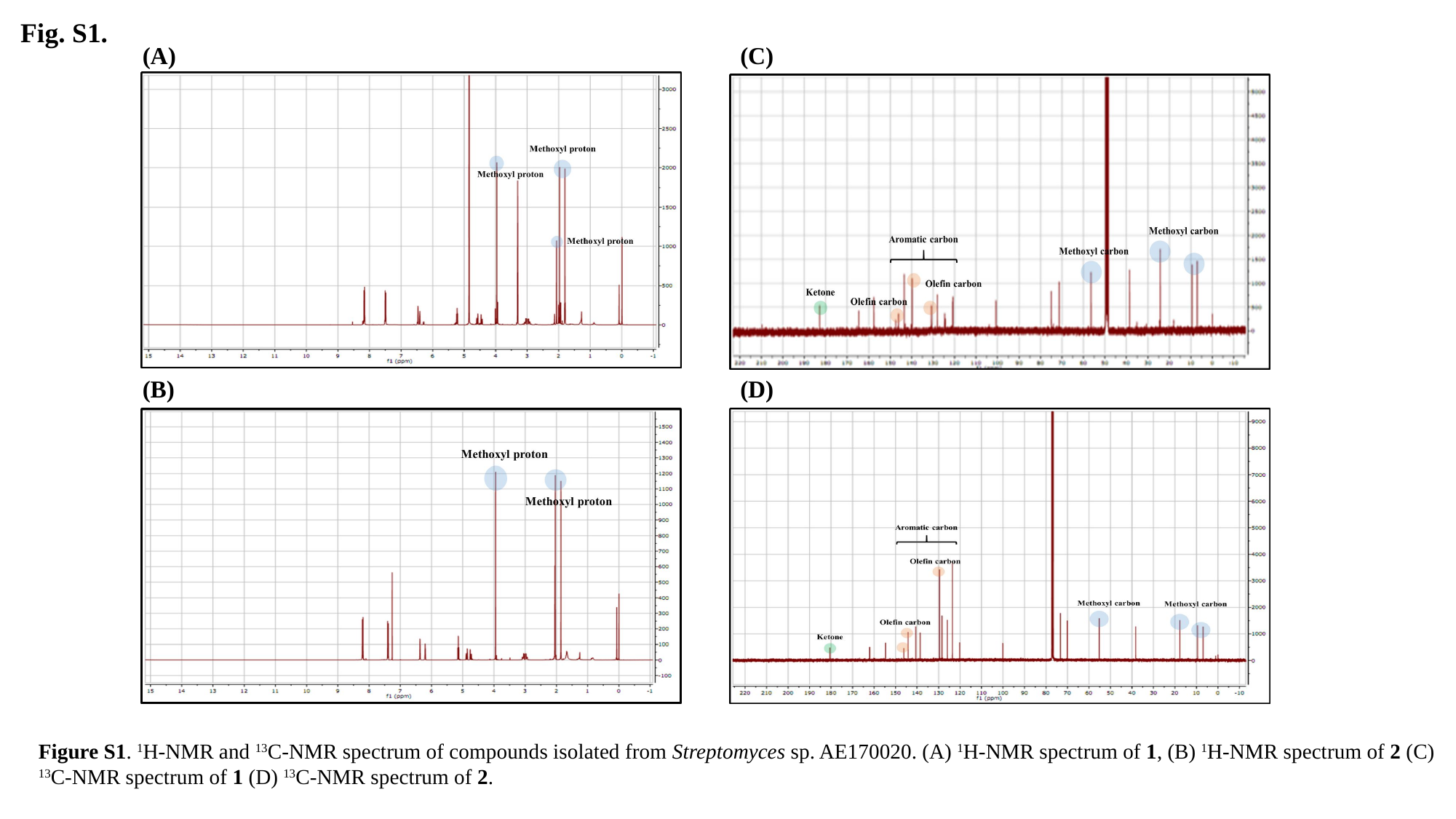

Fig. S1.
(A)
(C)
(B)
(D)
Figure S1. 1H-NMR and 13C-NMR spectrum of compounds isolated from Streptomyces sp. AE170020. (A) 1H-NMR spectrum of 1, (B) 1H-NMR spectrum of 2 (C) 13C-NMR spectrum of 1 (D) 13C-NMR spectrum of 2.

## Slide 2
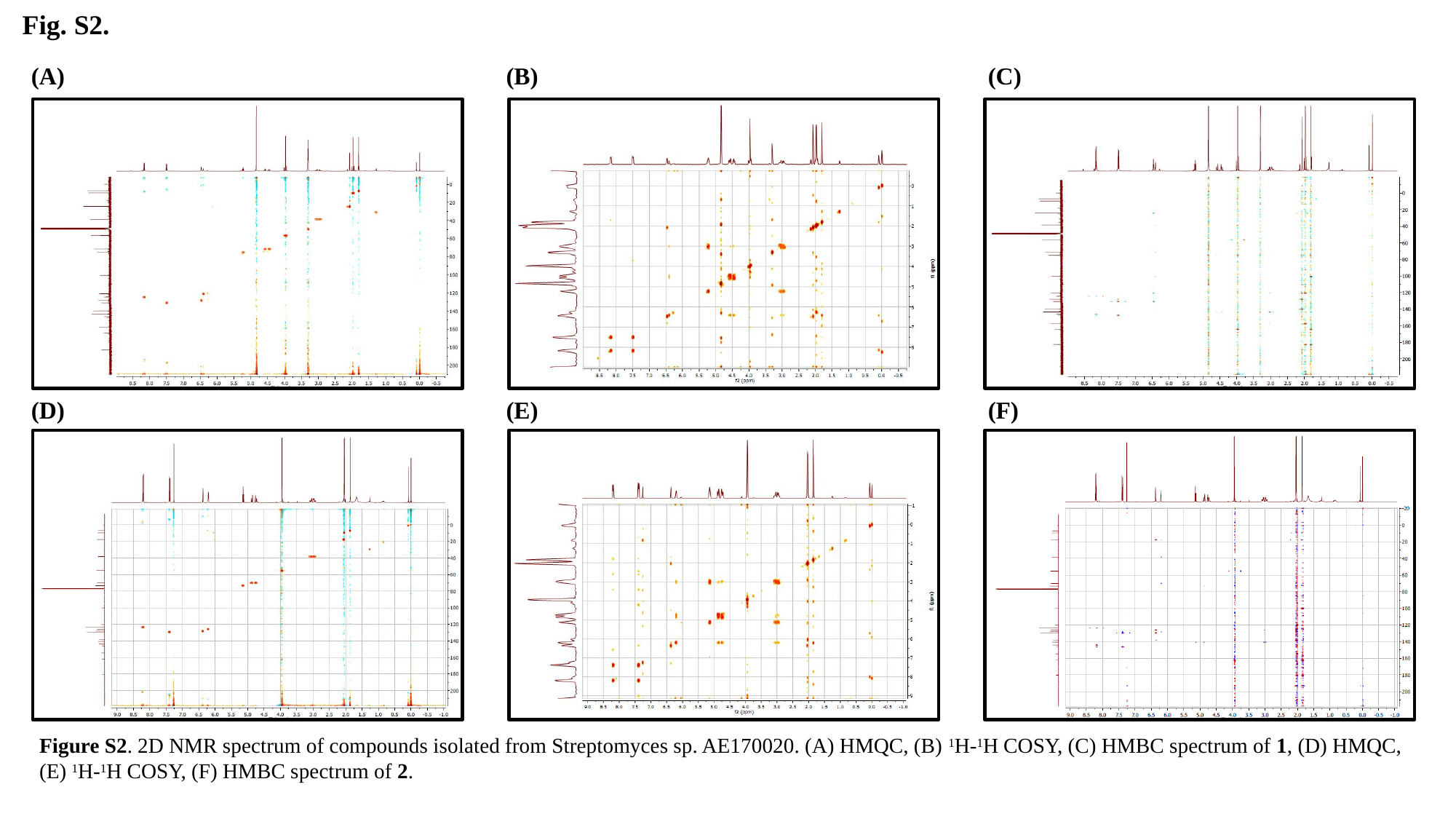

Fig. S2.
(A)
(B)
(C)
(D)
(E)
(F)
Figure S2. 2D NMR spectrum of compounds isolated from Streptomyces sp. AE170020. (A) HMQC, (B) 1H-1H COSY, (C) HMBC spectrum of 1, (D) HMQC, (E) 1H-1H COSY, (F) HMBC spectrum of 2.
